# ChromatoGate: A Tool for Detecting Base Mis-Calls in Multiple Sequence Alignments by Semi-Automatic Chromatogram Inspection

**DOI:** 10.5936/csbj.201303001

**Published:** 2013-05-08

**Authors:** Nikolaos Alachiotis, Emmanouella Vogiatzi, Pavlos Pavlidis, Alexandros Stamatakis

**Affiliations:** a Scientific Computing Group, HITS gGmbH, Heidelberg, Germany; b Institute of Marine Biology and Genetics, HCMR, Heraklion Crete, Greece; c Department of Genetics and Molecular Biology, Democritian University of Thrace, Alexandroupolis, Greece

**Keywords:** chromatograms, software, mis-calls

## Abstract

Automated DNA sequencers generate chromatograms that contain raw sequencing data. They also generate data that translates the chromatograms into molecular sequences of A, C, G, T, or N (undetermined) characters. Since chromatogram translation programs frequently introduce errors, a manual inspection of the generated sequence data is required. As sequence numbers and lengths increase, visual inspection and manual correction of chromatograms and corresponding sequences on a per-peak and per-nucleotide basis becomes an error-prone, time-consuming, and tedious process. Here, we introduce ChromatoGate (CG), an open-source software that accelerates and partially automates the inspection of chromatograms and the detection of sequencing errors for bidirectional sequencing runs. To provide users full control over the error correction process, a fully automated error correction algorithm has not been implemented. Initially, the program scans a given multiple sequence alignment (MSA) for potential sequencing errors, assuming that each polymorphic site in the alignment may be attributed to a sequencing error with a certain probability. The guided MSA assembly procedure in ChromatoGate detects chromatogram peaks of all characters in an alignment that lead to polymorphic sites, given a user-defined threshold. The threshold value represents the sensitivity of the sequencing error detection mechanism. After this pre-filtering, the user only needs to inspect a small number of peaks in every chromatogram to correct sequencing errors. Finally, we show that correcting sequencing errors is important, because population genetic and phylogenetic inferences can be misled by MSAs with uncorrected mis-calls. Our experiments indicate that estimates of population mutation rates can be affected two- to three-fold by uncorrected errors.

## Introduction

Genomic sequence analysis is an important task in bioinformatics and computational biology. Several applications, such as phylogenetic tree reconstruction or inference of population genetic parameters rely on genomic sequence data. Phylogenetic studies can be used to determine how a virus spreads over the globe [1] or to describe major shifts in the diversification rates of plants [2]. Population genetics can be used to infer demographic information such as expansion, migration, mutation and recombination rate in a population, or the location and intensity of selection processes within a genome.

In general, molecular sequence analyses involve a multitude of steps which depend on the specific scientific question at hand. Regardless of the concrete (downstream) steps in an analysis pipeline, the precise content and order of nucleotides in a stretch of DNA needs to be determined at the very beginning. The raw molecular sequence data is produced by DNA sequencing machines which analyze light signals that originate from flurochromes which are attached to nucleotides. Typically, the obtained raw sequences are provided as input to (multiple) sequence alignment programs. Once an MSA file has been generated, the analysis proceeds to address specific questions (e.g., phylogeny reconstruction or inferences of population genetic parameters). Thus, the quality of the initial MSA is of primary importance.

In some cases, only partially different or slightly erroneous MSAs can yield substantially different parameter values. For example, alignment errors can mislead the branch-site test [3] for positive selection such that it returns unacceptably high false positives [4]. Also, a slight over-representation of rare alleles may lead to inferring a population size expansion instead of obtaining a constant population size [5]. In phylogenetics, an uncorrected MSA may lead to biased estimates of tree topologies and branch lengths. In general, pseudo-polymorphic sites (sites that only appear to be polymorphic because of sequencing errors) can mislead downstream analyses.

A widely-used technique for reducing the number of sequencing errors and improving MSA quality consists in manually inspecting and visually verifying sequencer output based on the corresponding chromatogram files. This approach becomes increasingly tedious and error-prone as the number of sequences in the alignment as well as the length of sequences increases, since all sites need to be inspected individually. For each polymorphic site, users need to identify the exact chromatogram positions of polymorphic characters for that specific site and verify that they do not represent a pseudo-polymorphism.

We propose an approach for systematic detection and correction of sequencing errors in MSAs that relies on chromatogram data, henceforth denoted as the “CGF framework”. The goal is to generate corrected MSAs with the following property: each nucleotide in the MSA can be correctly mapped back to the corresponding chromatogram position by a few simple arithmetic operations. We denote this mapping property (or consistency) as CGP, and an MSA for which this property holds as CGA. Creating CGP-compliant alignments facilitates the detection and correction of possible sequencing errors in an MSA, because locating and identifying the potential error in the chromatogram trace signal now becomes straightforward. The user simply needs to approve or reject the respective base calls by visually inspecting the (automatically located) trace signal.

**Figure 1:**
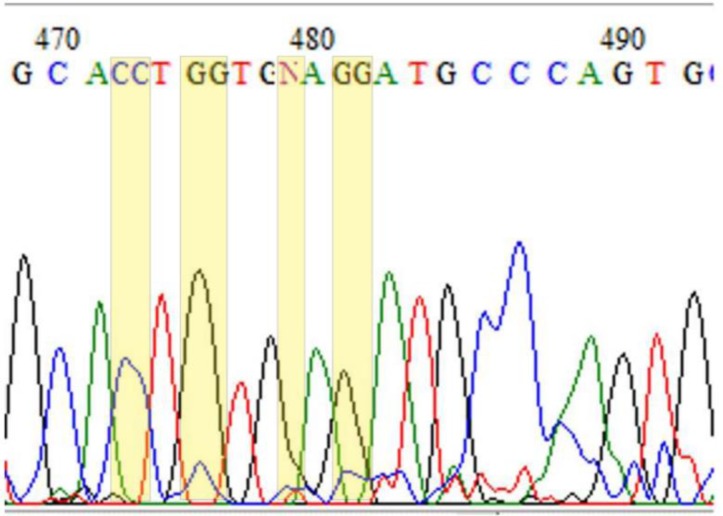
A chromatogram snapshot that shows base mis-calls and an undefined character at the highlighted positions.

Figure 1 depicts a chromatogram region that contains sequencing errors at the highlighted positions. At position 472, for instance, the slight expansion of the peak led to the insertion of the additional character C in the sequence. A similar peak expansion gave rise to duplication of the Gs at positions 475 and 481. Furthermore, the slightly shifted peaks at positions 478 and 480 resulted in the insertion of an undefined character N at position 479.

ChromatoGate implements the aforementioned CGF framework and thereby substantially simplifies the process of detecting sequencing errors and creating CGP-compliant MSAs. The development of ChromatoGate was motivated and guided by observing the workflow for reconstructing a phylogeny of 325 *Mullus surmuletus* sequences. While MSA tools (e.g., ClustalW [6], MAFFT [7], and MUSCLE [8]) required a few minutes and phylogeny reconstruction programs (e.g., RAxML [9] or MrBayes [10]) required a few hours to run to completion, the inspection of the MSA for detecting and correcting sequencing errors required several days. The tool is freely available for download at http://www.exelixis-lab.org/software.html.

There already exist some programs that provide a similar, but not identical, functionality to ChromatoGate. The Phred, Phrap, and Consed program suite [11, 12, 13–15] offers solutions for reviewing and editing sequence assemblies, such as trimming and assembling shotgun DNA sequence data. More specifically, Phred reads DNA sequencer trace files, associates them with the appropriate nucleotide base, and assigns a quality value to every base call. Thereafter, these quality values are used to trim the sequences. Phrap is intended for assembling shotgun DNA sequence data, and uses the entire sequence — not just the trimmed, high quality, part of a sequence. Phrap then constructs a contiguous sequence by merging the read segments with the highest quality, rather than by building a consensus sequence. Finally, the Consed/Autofinish tools aid the user in reviewing, editing, and finalizing Phrap-based sequence assemblies. Users can also select primers and templates, suggest additional sequencing reactions that shall be performed, and verify assembly accuracy.

DNA Chromatogram Explorer [16] is a dedicated interactive software for DNA sequence analysis and manipulation. Via an appropriate visualization method, it can trim low quality bases at either end of the sequences. The DNA Baser Sequence Assembler tool in Chromatogram Explorer can be used for DNA sequence assembly and analysis as well as for contig editing and mutation detection. An easy-to-use graphical editor is available to view and edit chromatograms, cut primers, assemble contigs, and reverse-complement sequences.

Sequencher 5.0 [17] (a commercial product by Gene Codes) is more similar to ChromatoGate in terms of functionality. The user can work with the chromatogram data and—at the same time—edit the corresponding raw sequences. Sequence edge trimming as well as tools for detection and annotation of polymorphisms are also available. Sequencher represents a useful software package that provides several tools for improving MSA quality, but the high software license cost ($ 1000) represents a substantial drawback.

ProSeq v3 (Processor of Sequences) [18] allows for the preparation of DNA sequence polymorphism datasets. It includes an internal relational database that links sequences to individuals and individuals to populations, thereby simplifying the analysis of datasets that contain multiple genes. Furthermore, it allows for visual inspection of DNA sequence chromatograms to correct base-calling and sequencing errors. Chromatogram quality checking is followed by assembly of individual sequence reads into longer contigs using Phred and Phrap (these are not included in ProSeq because of licensing issues). ProSeq v3 was developed for population genetic analyses and it also includes a tool for basic phylogenetic analysis that can construct and visualize neighbor-joining trees.

A significant difference between ChromatoGate and the aforementioned tools is that, in ChromatoGate, chromatogram editing is not implemented by visual alignment and chromatogram inspection, but via automatically generated reports. These reports only entail those nucleotides and associated chromatogram positions that require further inspection. By means of this pre-filtering, the user does not need to inspect the entire sequence base-by-base to correct sequencing errors, accelerating the error correction process.

## Experimental procedures

### Underlying Idea

**Figure 2:**
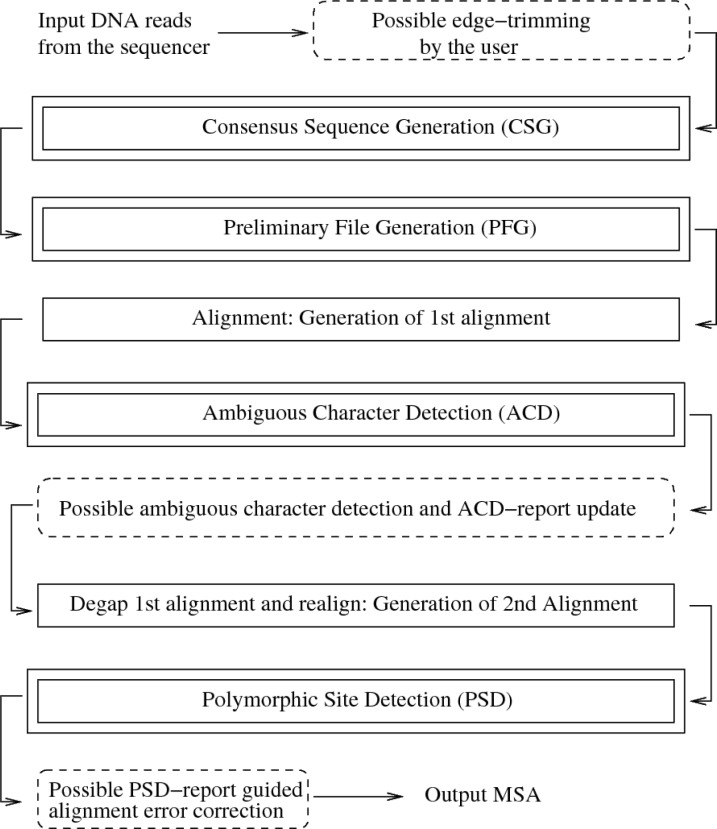
The steps of the CGF framework. The dashed-line boxes represent actions that need to be performed manually. The single-line boxes are used to refer to operations that can be performed by third-party software and the double-line boxes refer to ChromatoGate functions.

The goal of our tool is to generate CGP-compliant alignments with a minimal number of sequencing errors. This is achieved by a step-by-step alignment assembly and correction procedure which is illustrated in Figure 2. The steps outlined in Figure 2 are described in more detail below. Nonetheless, it should be evident that this workflow does not reflect the standard workflow of visual detection and manual correction of sequencing errors. The standard approach requires to consecutively correct all sequences prior to computing the MSA. Existing commercial (DNASTAR [19]) as well as open-source (Staden Package [20]) programs can be used for this purpose. They are able to identify weak-signal chromatogram peaks and report their positions to the user. Thereby, the user is not required to manually inspect all peaks in each chromatogram but only the indicated ones.

The semi-automatic ChromatoGate approach also requires the user to inspect a limited number of peaks in every chromatogram. The main idea is to inverse the process by scanning an initial—uncorrected— MSA for potential sequencing errors. Guided by this preliminary MSA, the user then only inspects those chromatogram peaks that form part of polymorphic sites in the alignment. Due to this inversed procedure for eliminating sequencing errors, ChromatoGate does not use base call quality information generated by programs such as Phred [11, 12].

In general, our approach treats every polymorphic site as a potential sequencing error. Usually, the sequencing error rate is not large (typically < 1%; [21]). Hence, base mis-calls will generate low-frequency polymorphisms, that is, polymorphisms that only occur in a few (typically 1 or 2) sequences of an MSA site. If a polymorphic site is not generated by a sequencing error but represents a true polymorphism, all characters at this site must have a clear, unequivocal chromatogram signal. If the corresponding peaks for a few characters (typically 1 or 2) at a polymorphic site are ambiguous, this can be attributed to a sequencing error rather than to a true polymorphism. Thus, the user only needs to visually inspect the chromatogram peaks of the comparatively small fraction of base calls that differ from the majority of bases at the site. Note that, ChromatoGate does not decide upon potentially erroneous base calls. Therefore, neither alignment sites are removed nor are base calls corrected. The tool assumes that a sequencing error most probably generates a low-frequency polymorphism in the alignment (see Figure 8). Evidently, removing all low-frequency polymorphisms from an alignment can not be desirable because it will generate a significant bias in the analysis (many genuine singletons will be removed along with the sequencing errors). To this end, ChromatoGate does not remove any low-frequency polymorphisms at all. Instead, it highlights them and maps them to the corresponding chromatogram peaks such that the user can assess if there is a sequencing error or a genuine singleton.

Note that, if recombination has occurred, it is likely that the number of base mis-calls in the MSA will be increased. ChromatoGate, however, cannot detect recombination. Therefore, if recombination has occurred, the ChromatoGate reports will be larger. Similar increase in the number of base mis-calls and thus in the size of the reports can be observed because of a very large nucleotide diversity in the MSA. In any case, the user can reduce the size of the reports by lowering the user-defined threshold, and thus determining in advance the amount of time he wishes to invest on the correction of the MSA.

When an existing MSA is extended by new sequences, this MSA-based correction process ensures that adding new sequences will not decrease the quality of an existing, curated reference MSA. Hence, ChromatoGate can not only be deployed for de novo MSA assembly, but also for MSA extension. We describe the CGF workflow and the corresponding ChromatoGate functions in more detail below.

### CGF Framework

#### Step1: Edge trimming

**Figure 3:**
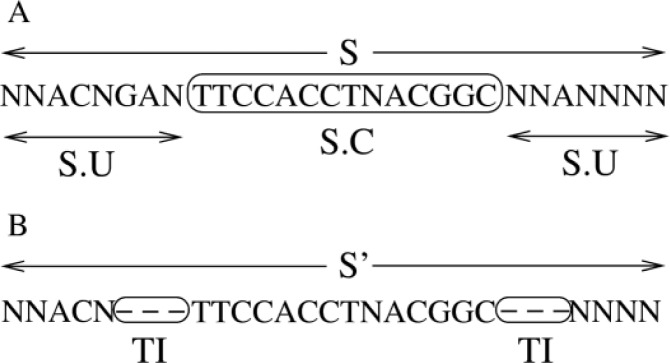
Example of the trimming process. A: The clean (S.C) and undefined (S.U) subsequences of S are shown. B: Trim-Indicators (TIs) of length 3 have been inserted in both S.U subsequences.

Typically, the chromatogram-based sequence S returned by a sequencer does not yield a clear signal over the entire sequence length. Thus, ambiguous subsequences S.U (Sequence.Undefined) that are characterized by a large number of undetermined characters appear at both ends of a sequence S. Typically, a clean subsequence S.C (Sequence.Clean) is located between S.U subsequences at either end. Only the clean subsequence entails nucleotides that can be identified by the sequencer with a high degree of confidence (Figure 3). Therefore, S.U subsequences must be trimmed to prevent biasing the downstream analysis. The responsibility to trim unreliable/undetermined subsequences rests with the user, because it is hard to design a sufficiently sensitive but not too sensitive tool for automatic trimming. Thus, for each sequence, the user needs to pass trimming position information (see below for details) to ChromatoGate.

Although several third-party tools for automated sequence trimming are available (e.g., the Lucy open-source code [22] using Phred scores or the Trim Ends function of the commercial Sequencher [17] package), deploying them in conjunction with ChromatoGate is not possible because base calls will be incorrectly mapped to corresponding chromatogram peaks. ChromatoGate uses the raw sequences (i.e., the exact position of each base call in the sequence) for mapping nucleotides to chromatograms. Hence, when edge-trimmed sequences are provided, this mapping will become inconsistent.

In Figure 3A we provide an example for sequence trimming. To preserve the CGP property, the S.U lengths for each sequence in a MSA must be known. Therefore, ChromatoGate keeps track of the initial sequence length, the length of trimmed subsequences, as well as the start and end positions of the S.C subsequences. User-driven trimming is implemented by Trim-Indicators (TIs) which are short sequences of one or more gaps that must be inserted by the user into the sequence to denote the beginning and end of the clean part of the (yet unaligned) sequence. The user will need to replace the last X characters of the left S.U by X gaps and the first Y characters of the right S.U by Y gaps. When no trimming is required, the user does not need to insert any gaps. In Figure 3B we provide an example for the trimming process (with X:= 3 and Y:= 3).

### Step 2: Consensus Sequence Generation

The second step (optional) consists of calculating consensus sequences for samples that have been amplified with forward and reverse primers. A DNA sequence is usually sequenced in both directions with a forward and a reverse primer (denoted as seqF and seqR respectively) when the entire length of the amplified gene product can not be obtained by a single primer. To obtain the full-length sequence, the reversed and complemented sequence seqRC of seqR needs to be aligned (matched) to seqF (note that, seqRC and seqF typically do not fully overlap). Finally, a consensus sequence is built for the pairwise alignment of seqF and seqRC (where seqF and seqRC overlap). ChromatoGate does not support consensus sequence generation from multiple pairs of forward and reverse reads at present. We will however, include this feature in the next release.

**Figure 4:**
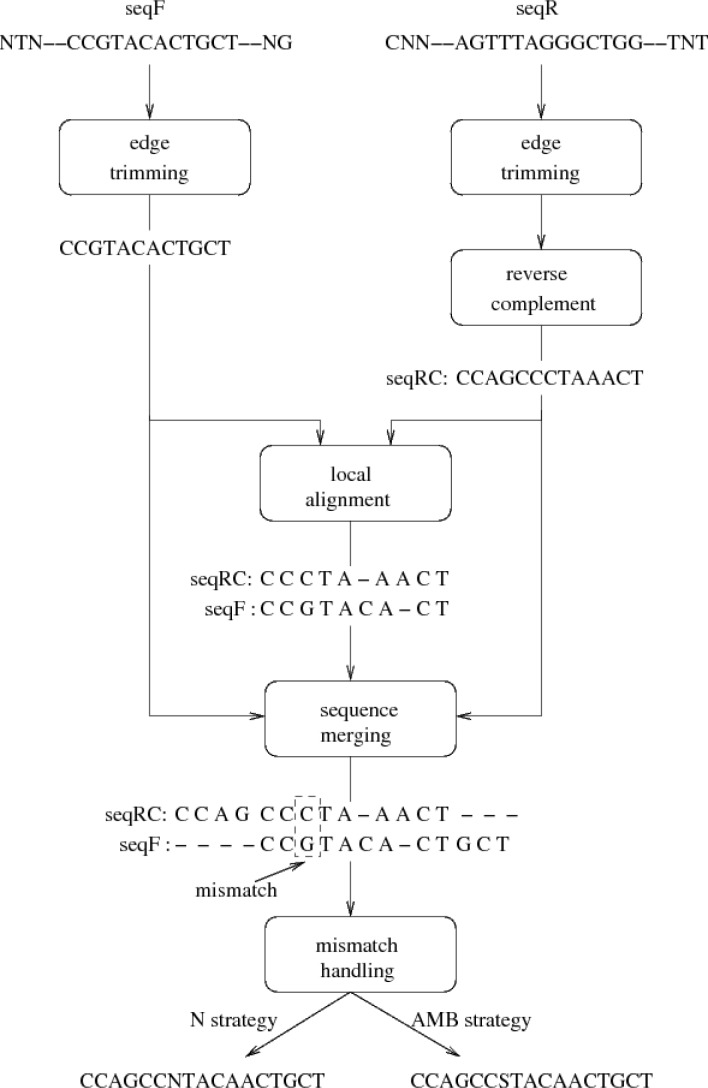
Steps of the Consensus Sequence Generation (CSG) procedure followed by ChromatoGate. The final consensus sequence consists of three subsequences: (i) the initial (non-overlapping with seqF) part of seqRC, (ii) the local alignment of seqRC and seqF for the part where they overlap, and (iii) the final (non-overlapping with seqRC) part of seqF.

The pairwise alignment and consensus sequence calculation in ChromatoGate is outlined in Figure 4. Initially, seqF and seqR are trimmed according to the TIs. Thereafter, seqR is reversed and complemented to obtain seqRC. Subsequently, ChromatoGate computes a local alignment of seqF and seqRC using a straightforward, naive implementation of the Smith-Waterman algorithm [23], since this step is not performance-critical. Two scoring matrices can be used for local alignment: (i) a default score matrix and (ii) a user-defined matrix. Note that, the Smith-Waterman algorithm locally aligns seqF to seqRC. Thus, we obtain an alignment in which seqF and seqRC only overlap partially. The trailing (non-overlapping) ends of seqF and seqRC are therefore attached again to the respective ends of the local alignment (see Figure 4). Finally, the sequences are checked for alignment mismatches to calculate a consensus sequence.

ChromatoGate offers two strategies for handling mismatches. It can either represent mismatches by the undetermined character N (N strategy) or by inserting the corresponding ambiguous character (e.g., S, R, Y, etc.; AMB strategy). In Figure 4, the alignment mismatch at site 7 can be handled by inserting an N (N strategy) or S (AMB strategy). In a subsequent step, ChromatoGate will provide the corresponding chromatogram positions of those mismatches, and allow the user to resolve them.

The consensus method handles mismatches as follows:

1. If the mismatch consists of a gap and a character, the character is used.
2. If the mismatch consists of two characters and none of them is an ambiguous character, then it will either be replaced by N (N strategy) or by the corresponding ambiguous character (AMB strategy).
3. If a mismatch consists of a character and an ambiguous character, then the (non-ambiguous) character is selected.

**Figure 5:**
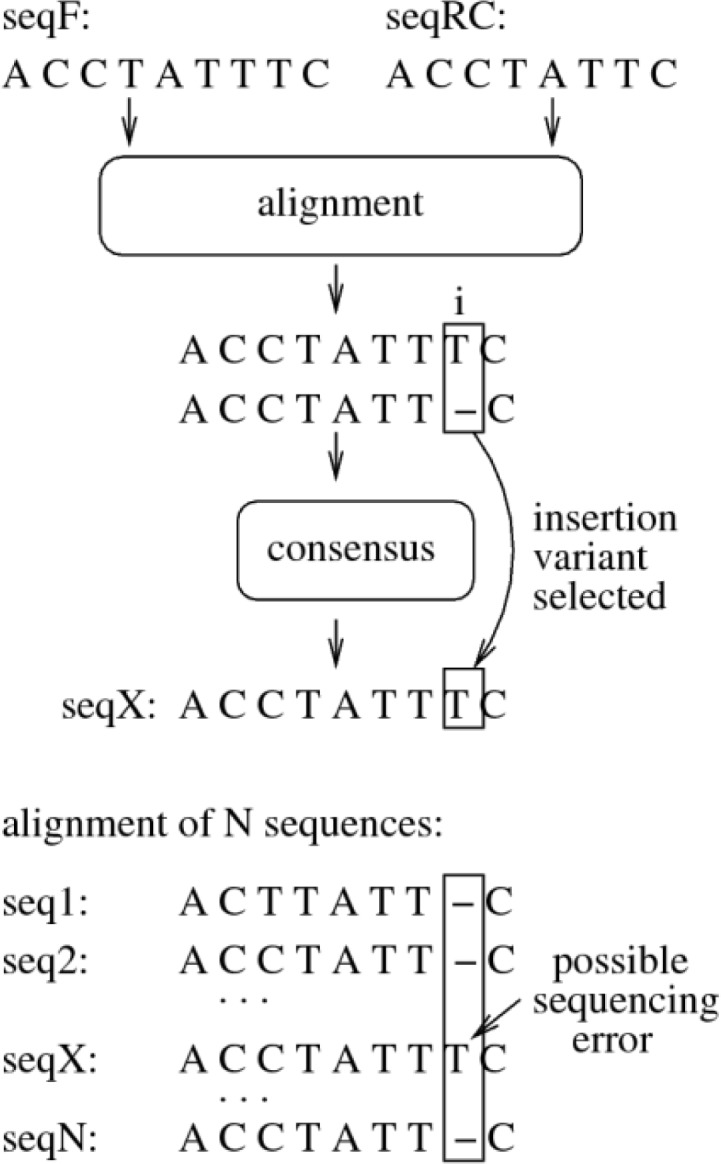
Detection of insertion/deletion sequencing errors in consensus sequences. During the generation of the consensus sequence seqX, the insertion variant is selected for the mismatch at position i. If character T of sequence seqF is actually the result of a sequencing error, the multiple sequence alignment will contain a gap-dominated site. The Polymorphic Site Detection (PSD) function will mark this site as one with a possible sequencing error.

Evidently, when extracting consensus sequences, the insertion variant is always selected (see Figure 5). This simplifies the detection of a possible sequencing error at a latter CGF step (see PSD – Polymorphic Site Detection). By selecting the insertion variant, it will generate alignment sites that are dominated by gaps and one or just a few characters. The characters of consensus sequences of gap-dominated sites are a consequence of selecting the constant insertion variant during consensus sequence generation. These ‘extra’ characters are usually generated by erroneously extended chromatogram peaks. The PSD step will classify these sites as sites with probable sequencing errors and report them to the user along with the positions of the corresponding chromatogram peaks.

When reconstructing consensus sequences based on a pairwise sequence alignment, additional information needs to be stored to maintain the CGP property. During pairwise alignment and consensus sequence generation, each character in seqF and seqRC can potentially be shifted and/or replaced by another character. ChromatoGate keeps track of all shift and replacement operations that are conducted prior to the computation of the multiple sequence alignment.

#### Step 3: Preliminary File Generation

After edge trimming, pairwise alignment, and consensus sequence generation, ChromatoGate generates a preliminary file of unaligned sequences in FASTA format (Preliminary File Generation - PFG). This preliminary file can then be used with any MSA program.

Apart from creating this FASTA file, ChromatoGate also maintains information for associating each nucleotide to the corresponding position in the respective chromatograms. Using the PFG-generated file for calculating MSAs ensures that it will be a CGP-compliant alignment regardless of the chosen MSA program. For every sequence in the PFG file and the MSA, ChromatoGate has maintained information on how each nucleotide was generated. This information can now be used to improve MSA quality.

#### Step 4: Ambiguous Character Detection

When the initial MSA has been calculated from the preliminary unaligned FASTA file, the Ambiguous Character Detection (ACD) function of ChromatoGate can be used to detect, inspect, and eventually correct all ambiguous characters in the MSA. For each ambiguous character, ChromatoGate automatically yields the correct position in the corresponding chromatogram and thereby substantially simplifies this process.

**Figure 6:**
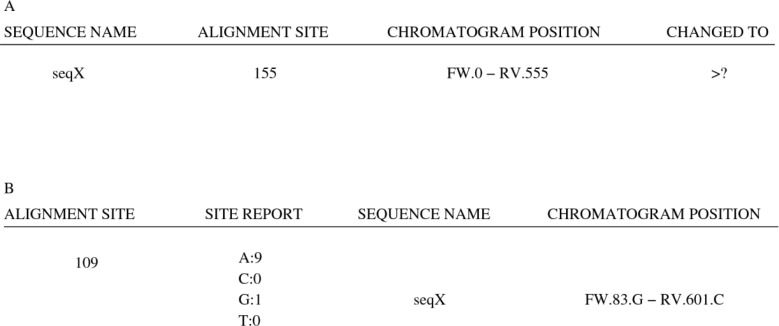
Example of an Ambiguous Character Detection (ACD) and Polymorphic Site Detection (PSD) report entries.

More specifically, ChromatoGate generates a report for every ambiguous character/site that allows the user to inspect the corresponding chromatogram positions. Erroneous ambiguous characters (as identified by visual chromatogram inspection) need to be corrected manually, and in this case, the respective ACD report entry must be updated accordingly. Figure 6A shows a typical ACD report entry for an ambiguous character. The ACD entry indicates that the ambiguous character was found at site 155 of sequence seqX in the MSA.

The chromatogram position field is of the form: FW.X - RV.Y, where X and Y correspond to the chromatogram positions in a forward and a reverse sequence respectively. For consensus sequences, both the F W.X and RV.Y fields are present while for forward or reverse sequences only the corresponding single field is shown. Therefore, from the ACD entry of Figure 6A one can deduce that the ambiguous character of seqX at site 155 was initially present in the reversed sequence and that it is located at position 555 of the respective chromatogram file.

If the user wants to correct this character he needs to update/edit the MSA (with an editor of his choice, e.g., BioEdit [24]) and update the corresponding ACD report entry by replacing the question mark (see Figure 6) with the new character in the “Changed To” field. Once all ambiguous characters have been inspected and potentially corrected the user needs to realign the sequences after de-gapping (removing the gaps from the previous alignment) them using the MSA editor. This new MSA should contain a lower number of polymorphic sites.

#### Step 5: Polymorphic Site Detection

A polymorphic site can either represent a “true” polymorphism or a base mis-call. The terms “pseudo- polymorphism” and “pseudo-polymorphic sites” are used synonymously to denote sites that appear to be polymorphic in an MSA due to one or more sequencing errors.

The Polymorphic Site Detection (PSD) function of ChromatoGate can be used for correcting pseudo-polymorphic sites. The PSD function implements a site-selection/-filtering mechanism with user-defined sensitivity. The selection sensitivity is set by a threshold which represents the maximum fraction of nucleotides that have to be different from the majority of nucleotides at a site such that the site is considered as polymorphic.

The output of the PSD function is a text file containing one entry for each polymorphic site in the MSA. Each entry contains the MSA site index, the nucleotide frequencies at that site, the names of those sequences whose nucleotides differ from the majority nucleotides at the site, and the corresponding chromatogram positions. As before, the PSD report can now be used to inspect the respective chromatogram files and correct errors. Figure 6B illustrates an entry of the PSD report. The report shows that site 109 of the MSA is polymorphic and contains 9 As, 1 G, and that the remaining characters, if any, are gaps. Nucleotide G belongs to the consensus sequence seqX and there are two corresponding chromatogram peaks: (i) the chromatogram peak that corresponds to character G at position 83 in the forward-primer-amplified sequence and (ii) the chromatogram peak that corresponds to character C at position 601 in the reverse-primer-amplified sequence. Once the PSD function has been applied to correct potential sequencing errors, the curated MSA will hopefully contain only a small number of ambiguous characters and sequencing errors.

## Results and Discussion

We assess the effect of uncorrected sequencing errors on phylogenetic and population genetics studies by means of simulations. Our simulated datasets consist of non-recombining genomic segments.

### Base mis-call simulation

We used two distinct settings (*errA*, *errB*) for incorporating sequencing error rates. Despite the fact that error rates typically vary significantly along a genomic segment [21], we simplified the error simulation process by assuming that base mis-calls are distributed uniformly along a genomic segment. There are two types of errors: (i) substitution errors that refer to erroneous calls of nucleotide states, that is, substitution of a base with an A, C, G, T, or N, and (ii) errors that lead to an insertion or deletion. We refer to the latter type of errors as frameshift errors. Frameshift errors are further sub-divided into nucleotide-state insertions (A, C, G, T, N), base deletions, or base-call extensions. For *errA*, the substitution error rate is set to 0.001 per base and the frameshift error rate is set to 0.0001. For *errB*, we set the substitution error rate to 0.01 and the frameshift error rate to 0.0005 (also see Figures 1 and 3 in [21]). The error rates are summarized in Table 1. Sequencing errors are introduced independently and uniformly for each sequence and for each base. Note that we neglect the effect of base mis-calls (especially frameshift errors) on the MSA by preserving homologous sites in the alignment. For example, if a base is inserted into a sequence, then a gap (-) is automatically inserted into all remaining sequences at the same position.

**Table 1:** Four error types were introduced in the simulated alignments. These errors correspond to i) misidentification, ii) insertion, iii) deletion, and iv) extension of a base. Two sets of error rates, *errA* and *errB*, were used in this study.

Error type	Error rate per bp (*errA*)	Error rate per bp (*errB*)
Misidentification	0.001	0.01
Insertion	0.0001	0.0005
Deletion	0.0001	0.0005
Extension	0.0001	0.0005

### Effect of mis-calls on phylogenetic tree reconstruction

We generated simulated DNA alignments to assess the effect of base mis-calls on phylogenetic tree reconstruction accuracy. To obtain realistic simulation parameters (GTR rates, *α*-shape parameter of the G model of rate heterogeneity [25], empirical base frequencies, reference tree) we initially conducted a single standard ML tree search with RAxML [9] on an empirical 500-taxon dataset [26] that has been frequently used for benchmarking phylogenetics software [27, 28, 29].

We then deployed INDELible [30] to generate 100 simulated datasets on the RAxML-based ML tree (using the ML model parameters as inferred for the empirical dataset) with a length of approximately 1,000 bp each. Indels were intentionally not simulated, since our goal was to explore the effects of sequencing errors on tree reconstruction accuracy. Hence, we avoided introducing further potential error sources (indels) that may also lead to decreased reconstruction accuracy to better identify the effects of base mis-calls. We then inserted base mis-calls into the 100 simulated alignments as described in Section “Base mis-call simulation” to generate an additional 100 simulated datasets with errors. Then, we conducted one ML tree search with RAxML (standard tree search, GTR model of nucleotide substitution, G model of rate heterogeneity) on the 200 simulated datasets (100 with and 100 without base mis-calls).

**Figure 7:**
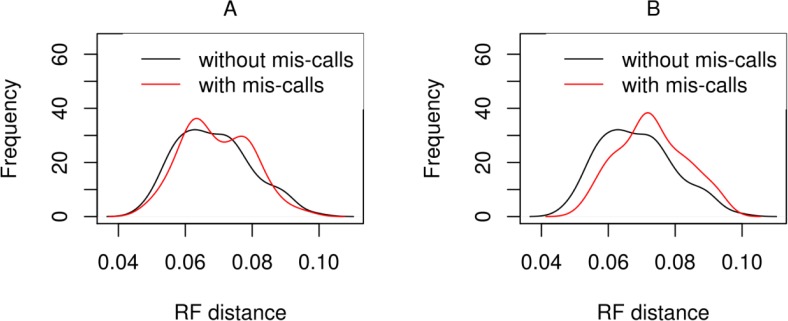
Robinson-Foulds (RF) distances distributions between inferred and real phylogenies when sequencing errors are absent (black line) or present (gray line) in the analysis. A) Error rates correspond to the values from the errA of Table 1. B) Error rates correspond to the values from the *errB* of Table 1. RF-distance quantifies the dissimilarity between two trees. Eliminating the sequencing errors from the analysis results in a statistical significant improvement of the similarity between the inferred and the true genealogy only for the higher error rates (*errB*), even though this difference is not very large.

Finally, we used the Robinson-Foulds (RF) metric [31] to determine the topological distance of the ML trees inferred on the simulated datasets with and without base mis-call errors to the respective true tree. As shown in Figure 7, base mis-calls significantly increase the RF distances (Kolmogorov-Smirnov statistic p-value = 0.0079) between the inferred ML tree and the true tree for the *errB* setting (substitution error probability: 0.01; frame change probability: 0.0005). However, RF distances only increased by 0.0045% on average for simulated alignments including sequencing errors. This may be attributed to the fact that maximum likelihood-based phylogenetic inference is relatively robust with respect to sequencing errors and noise. For smaller error probabilities (*errA* setting), the RF distances to the true tree are not significantly different on the datasets with and without errors (Kolmogorov-Smirnov statistic p-value = 0.527).

### Effect of mis-calls on population genetic parameters inference

We also examined the effect of base mis-calls on the inference of population genetic parameters. Our results indicate that, correcting for sequencing errors in the MSA is necessary. We simulated 2000 coalescent trees using Hudson's ms [32] tool. Each coalescent tree models the genealogy of 100 orthologous non-recombining genomic regions sampled from a constant population. We then used INDELible to simulate a 1-kb long, non-recombining single-gene dataset on each of the 2000 coalescent trees with the same empirical ML model parameters as above.

**Figure 8:**
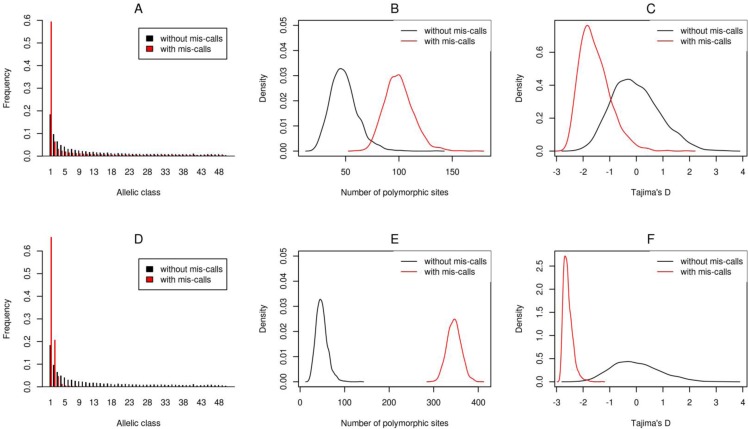
Biases in summary statistics that base mis-calls introduce in the analysis. At the upper panel we compare summary statistics calculated from datasets that contain no errors versus the datasets that contain errors from the set *errA* (see Table 1). For the bottom panel the error rates are described in *errB*. A and D: Base mis-calls shift the site frequency spectrum toward low-frequency polymorphisms. B and E: number of polymorphic sites, and C and F: Tajima's D. In all cases error rates introduce biases in the summary statistics. The biases are larger for higher error rates.

The branch lengths of the coalescent trees were scaled by a factor of 0.01, such that the population mutation parameter *θ = 4N*_*e*_*µ* is equal to 10 mutations per kilobase per *4N*_*e*_ generations, where *N*_*e*_ denotes the effective population size and *µ* the mutation rate per generation and per kilobase. We then introduced sequencing errors as described in Section “Base mis-call simulation”. MSAs were then converted to binary format (0,1), assuming an infinite site model [33]. This conversion is required because INDELible generates datasets for a finite site model, whereas the analysis assumes that the data follow the infinite site model. For every MSA column the most frequent nucleotide was transformed to state 0 and all remaining nucleotides to state 1. This infinite site model transformation is standard practice in population genetic data analysis and is justified by the small mutation rates. For all simulated datasets (with and without mis-calls) we calculated corresponding summary statistics such as to compare the respective distributions (Figure 8).

On the simulated datasets with errors, the Site Frequency Spectrum (SFS) shifts toward rare alleles (Figure 8A and 8D). Also, the number of singletons increases on average by a factor of 3 (compared to datasets without errors), even for small error rates (setting errA). The amount of polymorphic sites (as counted in the untransformed MSAs) increased by a factor of 2 for setting errA (Figure 8B) and by a factor of 7 for setting errB (Figure 8D) and Tajima's D [34] assumed extreme negative values (Figure 8C and 8F). Thus, base mis-calls dramatically affect estimates of population genetic parameters.

**Figure 9:**
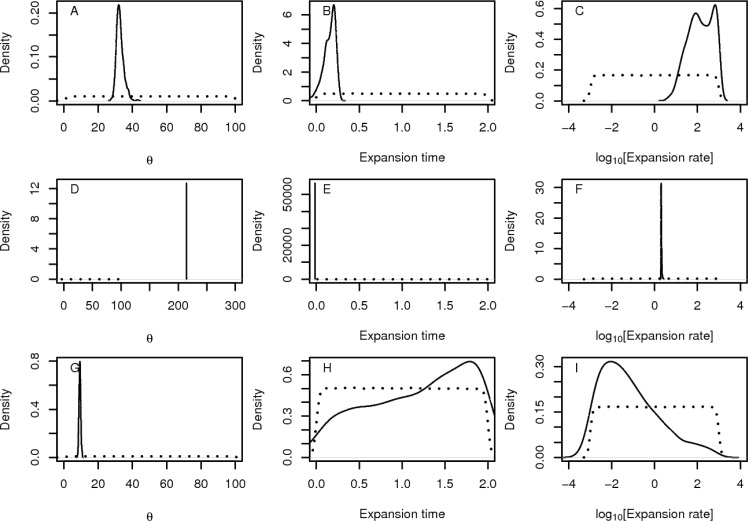
Base mis-calls bias the estimation of population genetics parameters. On the first two rows (A-F) the posterior distributions of parameters have been inferred from datasets that contain sequencing errors. At the bottom panel, sequencing errors have been removed. In figures A-C the error rates are given by the setA in Table 1, whereas Figures D-F error rates are described by the setB. The simulations implement a model of constant population size and θ = 10. Parameters were estimated using the ABC framework (see main text). In A, D, and G, we compare the posterior distribution of the population mutation parameter *θ*. B, E, and H show the posterior distributions of the time of population expansion, and C, F, and I show the expansion rate. Obviously, *θ* is overestimated when sequencing errors have been introduced. Furthermore, low-frequency alleles due to base mis-calls bias the analysis creating a signal of population size expansion. As expected, for MSAs with mis-calls, the estimated θ value is higher than the true value (Figures 9A, 9D, 9G) and a population expansion is detected (Figures 9B, 9C, 9E, 9F) when sequencing errors are present in the MSA. For small error rates (set *errA* in Table 1), the estimated θ value is 3 times higher than the real θ value. Furthermore, a recent and strong expansion is inferred (Figures 9B and 9C). For higher, but still realistic, error rates (set *errB* in Table 1), the inferred θ value is more than 20 times greater than the real θ value (Figure 9D). Additionally, a very recent expansion—that occurred just before the present—is inferred (Figure 9E). In contrast to this, analogous inference on the reference dataset without base mis-calls did not yield significant deviations from the true, simulated parameter values (Figures 9G, 9H, 9I). The estimated θ value is 10 Figure 9G), the maximum *a posteriori* rate of expansion is -2.23, and the inferred time of expansion amounts to approximately 1.8 N_e_ generations. In other words, a very old and weak expansion has been inferred on the reference dataset without base mis-calls.

We also simulated a multi-gene dataset from a constant population using msABC [35], comprising 20 independent, non-recombining genes with a length of 1 kb. This dataset represents the reference alignment without base mis-calls. Thereafter, we introduced errors as before to create a reference dataset with base mis-calls. These datasets were used to infer population parameters using an Approximate Bayesian Computation (ABC) method as implemented in the ‘abc’ package [36] of the R programming language for statistical computing [37]. We sampled 1,000,000 candidate parameter vectors and used them to simulate multi-gene datasets with msABC [35]. The simulated and reference datasets were summarized using the average values of θ_π_ [38], θ_W_ [39], Z_NS_ [40], and H [41]. For each dataset we retained the 1,000 most similar simulations, based on the distance between the summary statistics of the simulated and the reference datasets. Then, we assessed the bias introduced by mis calls on estimates of the population mutation rate *θ* and past population size changes.

### Saving Analysis Time with ChromatoGate

To obtain an estimate of potential analysis time savings, EV tested ChromatoGate on 325 *Mullus surmuletus* sequences from the D-loop region with a length of 350 base pairs. These sequences had already been manually corrected by EV within 3 working days. EV repeated the error detection and correction procedure using ChromatoGate in 6 hours. The ChromatoGate-based correction detected more sequencing errors within a significantly smaller amount of time.

## Conclusions

We have presented the freely available and easy-to-use software ChromatoGate that allows for rapidly identifying and correcting base mis-calls as generated by capillary and gel-based sequencers. It also allows for aligning and merging forward and backward sequences and computing respective consensus sequences. The key feature of ChromatoGate is that it maintains meta-data that allows the user to “go back” and inspect chromatogram peaks at any point of the MSA assembly process and correct potential errors. A purely empirical assessment of the time saving that can be achieved by using such a semi-automatic tool indicates that biologists can save approximately one order of magnitude of office time by deploying ChromatoGate.

We also address the more fundamental question whether base-mis calls need to be corrected at all for phylogenetic and population genetic analyses by means of simulated data experiments. Our experiments indicate that correcting for base mis-calls in MSAs used for phylogenetic analyses is not absolutely necessary. However, correcting for base mis-calls in population genetic analyses, which rely on inferring many parameters based on a substantially smaller number of evolutionary events than in phylogenetic analyses, appears to be absolutely necessary. If one does not correct for base mis-calls to get those few mutations right in population genetic analyses, parameter estimates can deviate significantly from their true values.
